# Epidemiology of Ectoparasites (Ticks, Lice, and Mites) in the Livestock of Pakistan: A Review

**DOI:** 10.3389/fvets.2021.780738

**Published:** 2021-12-16

**Authors:** Ali Muhammad, Rida Bashir, Majid Mahmood, Muhammad Sohail Afzal, Sami Simsek, Usman Ayub Awan, Mobushir Riaz Khan, Haroon Ahmed, Jianping Cao

**Affiliations:** ^1^Department of Zoology, University of Poonch Rawalakot, Azad Jammu and Kashmir, Pakistan; ^2^Department of Life Sciences, Faculty of Science, University of Management and Technology (UMT), Lahore, Pakistan; ^3^Department of Parasitology, Firat University, Elâzig, Turkey; ^4^Department of Medical Laboratory Technology, The University of Haripur, Haripur, Pakistan; ^5^School of Environmental Science, Charles Sturt University, Albury, NSW, Australia; ^6^Department of Biosciences, COMSATS University Islamabad (CUI), Islamabad, Pakistan; ^7^Chinese Center for Disease Control and Prevention (Chinese Center for Tropical Diseases Research), National Institute of Parasitic Diseases, Shanghai, China; ^8^Key Laboratory of Parasite and Vector Biology, National Health Commission of the People's Republic of China, Shanghai, China; ^9^WHO Collaborating Center for Tropical Diseases, Shanghai, China; ^10^Chinese Center for Tropical Diseases Research, The School of Global Health, Shanghai Jiao Tong University School of Medicine, Shanghai, China

**Keywords:** epidemiology, ectoparasites, ticks, mites, lice, livestock, Pakistan

## Abstract

Ectoparasites, including lice, ticks, and mites, inhabit the host skin and depend on their host for sustenance, maturation, and multiplication. Among these, ticks are more prevalent in various regions of Pakistan because of favorable climatic conditions, lack of awareness of livestock keepers' regarding ectoparasite infestation rate, insufficient veterinary services, and inadequate control measures. Ectoparasitic infestation is a primary threat to cost-effective livestock production by damaging skin and transmitting multiple diseases between animals. This review aimed to determine the infestation rates of various ectoparasites in cattle, buffaloes, sheep, goats, camels, equids and to ascertain the prevalence and epidemiology of ectoparasites in different regions of Pakistan. This review could be useful in devising prevention and control strategies and identifying the risk factors associated with ectoparasites to enhance animal productivity. It provides directions for veterinary schools, researchers, and organizations aiming to collaborate with neighboring countries to eradicate these parasites. Future studies could support working veterinarians and administrators and contribute to human well-being.

## Introduction

Pakistan is an agricultural country with 75% of its population involved directly or indirectly in agriculture. It is the second-largest sector, providing 21.2% of the gross domestic product (GDP) and employment to 45% of the labor force (1). Livestock, the “spine of the Pakistan agricultural economy,” are in danger due to the huge numbers of ecto- and endoparasites (2, 3), and the costs of the control measures could have a serious economic impact on the livestock and dairy industries (4). The livestock sector is an integral part and the basis of the rural economy (5) as more than 70% of the population resides in rural regions (6). Domestic mammals contribute 53.2% of the agricultural worth and 11.4% of the overall GDP. Buffaloes produce about 68% of the milk in Pakistan, while 27% is produced by cattle, and 5% by sheep, goats, and camels (7).

Livestock is the main source of energy, food, raw materials, and compost for crops. Consequently, it is not surprising that livestock, particularly the dairy industry, have risen as an important economic source and a trademark for the agri-business in dairy, meat, and numerous other products (8). Cows and buffaloes are a key source of animal proteins, and their products, such as bones, skins, and products made from their components, are of great importance for humans (9). Cattles are used as a source of meat, milk, and other dairy products; however, ticks harm their skin and hinder meat and milk production (10). Goats invest an impressive quantity of vital proteins in their struggle against a diversity of ectoparasites, and frequently transfer a range of pathogens (11). Sheep have great social and economic importance as they are used for cultural merriments and religious sacrifices to counter crop failure (12). Parasite infection places a major restriction on profitable dairy production (13).

Ectoparasites, including lice, ticks, mites, fleas, are organisms that inhabit the skin or skin surface of another organism (the host) for several days and could be detrimental as they depend on their host for sustenance, maturation, and multiplication. As “a principal blockage to the growth of animals,” ectoparasites play a vital role in the spread of specific pathogens (14). For example, ticks and mites are the vectors of many bacterial, viral, rickettsial, and protozoal diseases, some of which are zoonotic (15).

Ectoparasitic infestation poses the greatest threat to cost-effective livestock production (16). Ectoparasites are involved in mechanical damage, anemia, loss of condition, irritation, allergic reaction, toxicosis, morbidity, and mortality. Indirect effects of ectoparasites consist of transmission of pathogens that cause babesiosis, theileriosis, anaplasmosis, and more (17). Some parasites even cause diseases in humans when the protection measures are ignored (11).

Among the ectoparasites, ticks have been recognized as a disreputable threat due to the severe irritation, allergy, and toxicosis they cause, and diseases like babesiosis, theileriosis, and anaplasmosis they transmit (18). Ticks are potential disease vectors and act as reservoirs of certain infectious agents (2). They are vectors of several pathogenic microorganisms, including viruses, bacteria, spirochetes, rickettsia, and protozoans (19), acting as a cause of morbidity in livestock (20). Livestock are also affected by other tick damages, including tick-bite abscesses, irritation, dermatophilosis, and blood loss with its detrimental stressful effect on the animals (21). Ticks are obligate blood-sucking parasites (3), with the hard ticks having an outstanding medical and veterinary importance (22).

Mange mites were blamed for great economic losses due to the damages they cause to the skin and wool, anemia, poor body condition, and decreased milk and meat production and growth rate (23). Louse infestation is the base of reduced hide and skin features which influence tanning industry and ruins country's economy, about 1.9 to 94% of louse infestation in cattle and buffaloes raised under different management system (24, 25). Mites cause severe losses due to rejection skin, loss of production, anemia, and death when found in large numbers (25).

Livestock sector is backbone of the country like Pakistan and till date no up to date information on ectoparasites were available. That why the current review was designed on the epidemiology of ectoparasites (Ticks, lice and mites) in livestock of Pakistan.

## The Review Protocol

This review was conducted following the Preferred Reporting Items for Systematic Reviews and Meta-Analyses (PRISMA). All available published research articles on ectoparasites in livestock were considered. The various review steps included literature search, inclusion and exclusion criteria for relevance to the topic, and extraction of relevant data to achieve the study objective.

## Literature Search

The literature search included all studies published during 2002–2020 on livestock ectoparasites in Pakistan. The search used Google Scholar, PubMed, NCBI, ResearchGate, and Web of Science. Keywords used for the search were ectoparasites, ticks, lice, mange mites, flies, fleas, small ruminants, livestock, sheep, goat, cattle, buffalo, horse, camel, donkey, prevalence, domestic animals, risk factors, livestock diseases caused by ectoparasites, tick and lice infestations, the incidence of ectoparasites, threats to the dairy sector, theileriosis, anaplasmosis, cattle tick, and Pakistan. Various keyword combinations were used, and the full text of selected articles and their reference lists were screened to relevant articles.

**Figure 1:**
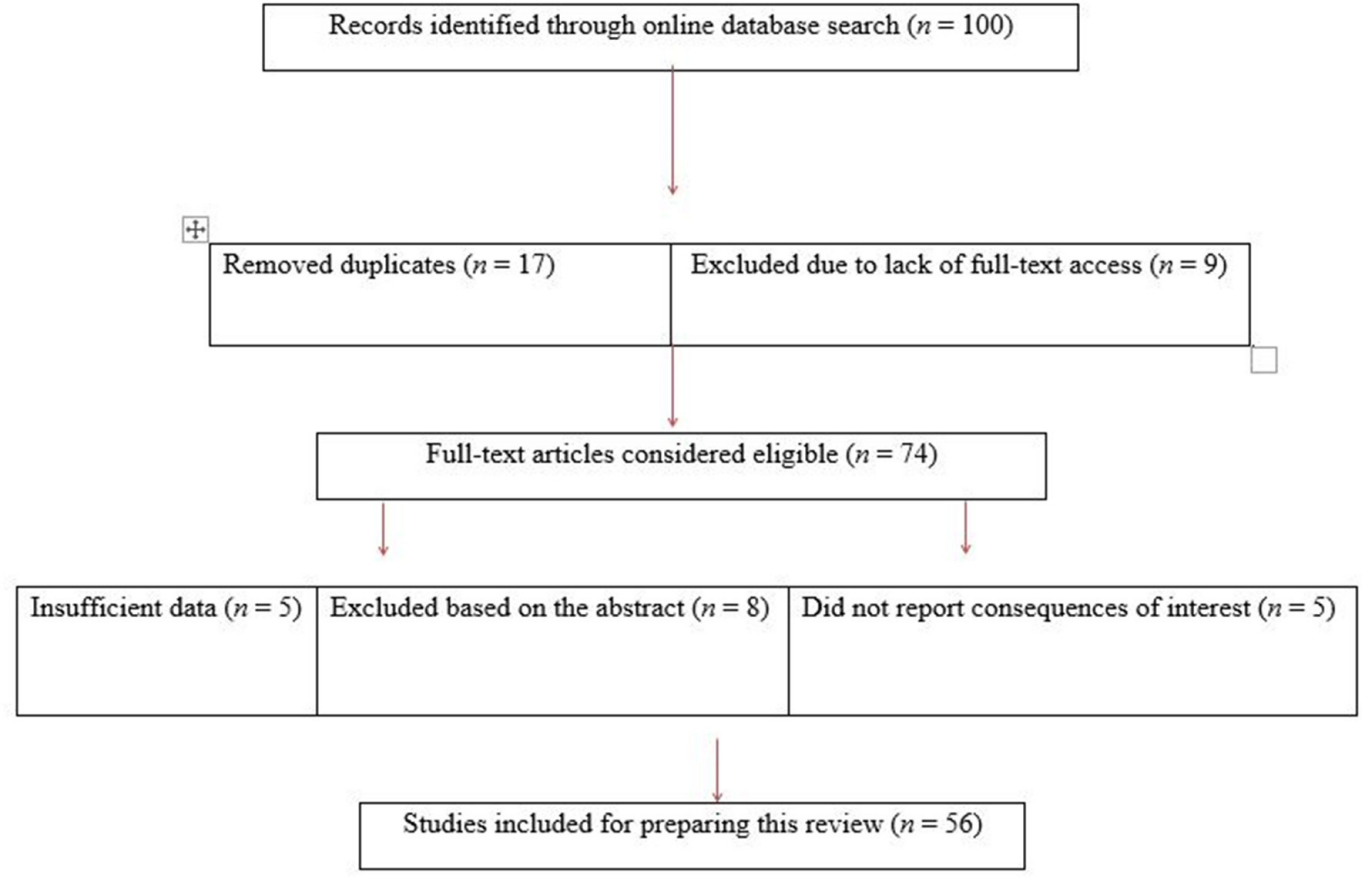
An overview of the study selection process.

We identified 100 articles related to animal ectoparasites in Pakistan for this review. However, 17 articles were excluded due to duplication. We included 56 articles in this review while 44 articles were excluded. The literature assessment and selection process is illustrated in Figure 1.

## Results and Discussion

**Figure 2:**
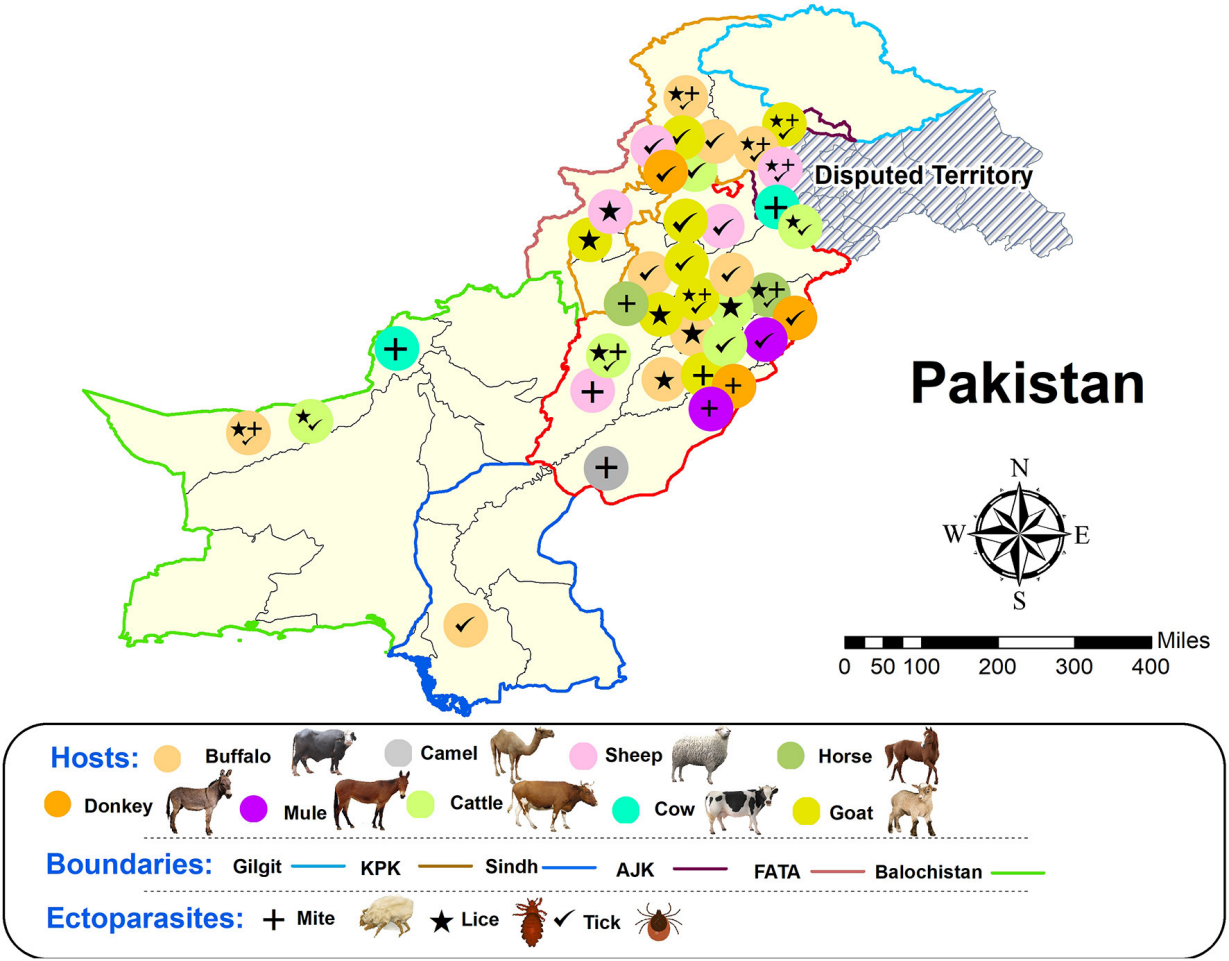
Distribution of ectoparasites among different hosts from different provinces of Pakistan. Animal/Insects icons for reference are taken from different web sources.

An attempt was made to compile all available published research articles on livestock ectoparasites in Pakistan. Prevalence was estimated as the number of host specie infected by at least one parasite divided by the total number of host specie examined for parasites. The distribution of ectoparasites among different host is shown in Figure 2. Data on the prevalence of livestock ectoparasites was collected from the included studies.

### Prevalence of Lice in Pakistan

Lice are small wingless parasitic insects on animals that suck their blood. Louse infestation reduces hide and skin features, influencing the tanning industry and having a harmful economic impact. Louse infestation was found in 1.9–94% of the cattle and buffaloes raised under various management systems (24).

The prevalence of louse infestations varies between the regions of Pakistan and depends on the region studied. Fourteen studies investigated louse infestation in cattle, sheep, horses, buffaloes and cattle calves in Pakistan, and four studies focused on lice in cattle or buffaloes. Details of source, host, area, lice, and estimated prevalence are presented in Table 1. Data of reported prevalence estimates for lice infestation varied between species, as the prevalence in goats differed from that in sheep, cattle, and buffaloes.

**Table 1:** Characteristics of key studies conducted on lice infestations of livestock in Pakistan.

**Area/city**	**Study year**	**Longitude**	**Latitude**	**Host**	**Sample size**	**Prevalence**	**References**
Dir district	2002	72.0468° E	35.3356° N	Buffalo calves	118	34.75%	(26)
Lahore	2005	74.3587° E	31.5204° N	Horse	84	0%	(27)
Faisalabad	2004–2005	73.1350° E	31.4504° N	Cattle and buffalo	1,200	Cattle, 24%; buffalo, 18%	(28)
Quetta	2008	66.9750° E	30.1798° N	Cattle and buffalo	790	Cattle, 7.17%; buffalo, 9.84%	(29)
Punjab	2006–2007	72.7097° E	31.1704° N	Cattle	300	36.3%	(30)
Multan	2008	71.5249° E	30.1575° N	Buffalo	100	92%	(31)
Dera Ghazi Khan	2008	70.6455°E	30.0489° N	Cattle	300	5%	(32)
Quetta	2006	66.9750° E	30.1798° N	Cattle and buffalo	1,580	Cattle, 38.3%; buffalo, 41.2%	(9)
Punjab	2011–2012	72.7097° E	31.1704° N	Cattle	3,864	23.23%	(17)
Toba Tek Singh	2011–2012	72.652359° E	30.894875° N	Goat	4,020	9.58%	(11)
Rawalakot (AJK)^*^	2014–2015	73.7654° E	33.8584° N	Cattle and buffalo	200	0% in both	(24)
Muzaffarabad (AJK)^*^	2015	73.4769° E	34.3551° N	Sheep, goats, cattle, and buffalo	100	41% overall (Lice, ticks, and mites)	(25)
Kotli, (AJK)^*^	2015	73.9007° E	33.5008° N	Buffaloes, sheep, and goats	200	26% overall (Lice and ticks)	(16)
Karak	2018	71.0914° E	33.1105° N	Sheep and goats	75	Sheep, 20%; goats, 13.3%	(33)

* *Azad Jammu and Kashmir*.

#### Prevalence of Lice in Buffaloes

The prevalence of lice in buffaloes ranged between 0 and 92%. Lice infestation in the buffaloes of Multan (31) was 92%, considerably higher than the 0% prevalence reported in the cattle and buffaloes of Azad Jammu and Kashmir (24). In 2002, 118 buffalo calves were investigated in the Dir District, and 34.75% were found to harbor lice (26). A study in Faisalabad reported in 2006 that about 18% of the buffaloes were infested with lice, higher than the 9.84% reported in Quetta (28, 29). The overall highest prevalence of lice, ticks, and mites recorded during the month of July, 2008 in the sheep, goats, buffaloes, and cows of the Muzaffarabad District, Azad Jammu and Kashmir was 41% (25), while a study from 2016 (16), recorded an overall louse and tick prevalence of 7.5% in the goats, buffaloes, and sheep of Kotli, Azad Jammu and Kashmir (Table 1).

#### Prevalence of Lice in Goats

The range of louse infestation was from 9.58% in the goats of the Toba Tek Singh District (11) to 13.3% in the goats of Karak in Khyber Pakhtunkhwa (33).

#### Prevalence of Lice in Sheep

A louse prevalence of 20% in the sheep of Karak, Khyber Pakhtunkhwa was reported in 2008 (33). An overall louse and tick prevalence of 26% was reported in 2016 in the goats, buffaloes, and sheep of Kotli, Azad Jammu and Kashmir (16) and an overall prevalence of 41% in sheep, goats, buffaloes, and cows was reported in the Muzaffarabad District, Azad Jammu and Kashmir (25).

#### Prevalence of Lice in Cattle

The louse prevalence in cattle was in the range of 0–38.3%. A 0% prevalence was reported in Rawalakot, Azad Kashmir in 2015 (24), 5% in DG Khan (32), 23.3% in Punjab (17) and 38.3% and 7.17% was reported in Quetta (9, 29), 24% in Faisalabad (28).

#### Prevalence of Lice in Horses

Of the 14 studies on lice, only one reported about horses. A study from 2005 (27) examined 84 horses but found no lice infestation, i.e., a prevalence of 0% (Table 1).

### Prevalence of Mites in Pakistan

Any of the numerous small acarid arachnids that often infest animals could cause inflammation and loss of hair. Mange mites are an important ectoparasite of sheep (34), resulting a severe economic impact among livestock (34). The prevalence of mites varied between parts of Pakistan, as shown in Table 1.

#### Prevalence of Mites in Sheep

The prevalence of mite infestation in sheep ranged from 6 to 17% in several studies. In Punjab, prevalence was 11.37% (34), 17% (12), and 6% (23). In 2016, overall prevalence (louse and tick) was 26% among sheep, goats and buffaloes from AJK (16). Details are shown in Table 2.

**Table 2:** List of key studies conducted on mite infestations of livestock in Pakistan.

**Area**	**Study year**	**Longitude**	**Latitude**	**Host**	**Sample size**	**Prevalence**	**References**
District of Dir	2002	72.0468° E	35.3356° N	Buffalo calve	118	11.86%	(26)
Lahore	2005	74.3587° E	31.5204° N	Horse	84	29.76%	(27)
Lahore	2005	74.3587° E	31.5204° N	Horse	48	53.33%	(35)
Dera Ghazi Khan	2007	70.6455° E	30.0489° N	Sheep	400	6%	(23)
Quetta	2008	66.9750° E	30.1798° N	Cow and buffalo	790	5.19 and 4.92%	(29)
Punjab	2008	72.7097° E	31.1704° N	Cattle	300	4%	(30)
Dera Ghazi Khan	2008	70.6455° E	30.0489° N	Cattle	300	8%	(32)
Punjab	2013	72.7097° E	31.1704° N	Horse, donkey, and mule	450	Overall prevalence (1.5%)	(36)
Punjab	2011–2012	72.7097° E	31.1704° N	Cattle	3,864	4.34%	(17)
Toba Tek Singh	2011–2012	72.652359° E	30.894875° N	Goat	4,020	3.23%	(11)
Punjab	2013–2014	72.7097° E	31.1704° N	Camel	1,489	11.28%	(37)
Toba Tek Singh	2010–2011	72.652359° E	30.894875° N	Sheep	800	11.37%	(34)
Dera Ghazi Khan	2013–2014	70.6455° E	30.0489° N	Sheep	500	17%	(12)
Multan	2016	71.5249° E	30.1575° N	Goats	200	35.5%	(38)
Muzaffarabad (AJK)	2015	73.4769° E	34.3551° N	Buffalo, cow, sheep, and goat	100	Overall prevalence (Mites, ticks and lice) of 41%	(25)
Cholistan	2010–2011	71.5724° E	28.5062° N	Camel	450	Overall prevalence (Tick and mite/fly) of 55.56%	(39)

#### Prevalence of Mites in Buffaloes

The highest mite prevalence of 11.86% was recorded in buffalo calves in 2002 in the Dir District (26), and the lowest (4.92%) was reported in the buffaloes of Quetta (29).

#### Prevalence of Mites in Camels

Of the 16 studies on mites as ectoparasites, only two reported about camels. In 2015, 1,489 camels were examined for a mite infestation, recording an infestation rate of 11.28% from three (North, south and western) parts of Punjab (37). In a study in the Cholistan area, 55.56% of the camels were infested by these ectoparasites (including ticks and lice) (39).

#### Prevalence of Mites in Equids (Horse, Mule, and Donkey)

Of the 16 studies on mites, three reported mite prevalence in horses, mules, and donkeys.

Two studies reported a prevalence of 53.33% (35) and 29.76% (27) in the horses of Lahore. A study from 2013 (36) recorded an overall prevalence of 1.5% in the horses, donkeys, and mules of Punjab.

#### Prevalence of Mites in Goats

Only two studies reported the prevalence of mite infestations in goats. A study in Toba Tek Singh recorded a prevalence of 3.23% (11), while in a study in Multan, 35.5% of the goats were found to be infested with mites (12).

#### Prevalence of Mites in Cattle

Of the 16 studies published on mites as ectoparasites of domestic animals in Pakistan, four investigated the prevalence in cattle alone, and one recorded an overall prevalence in several animal species, including cows. The range of mite infestation in these studies was 4–8%. A study from 2008 in Quetta (29) observed that 5.19% of the cows were infested with mites. Three studies on cattle in Punjab reported prevalence rates of 5% (32), 4% (30), and 4.34% (17).

### Prevalence of Ticks in Pakistan

The prevalence of ticks infestation in different livestock species as shown in Table 3.

**Table 3:** List of key studies conducted on tick infestations of livestock in Pakistan.

**Area**	**Study year**	**Longitude**	**Latitude**	**Host**	**Sample size**	**Prevalence**	**References**
District of Dir	2002	72.0468° E	35.3356° N	Buffalo calve	118	5.08%	(26)
Lahore	2005	74.3587° E	31.5204° N	Horse	84	0%	(27)
Punjab and KPK	2007	72.7097° E 72.3311° E	31.1704° N 34.9526° N	Cattle, buffalo, sheep, and goat	18,000	Cattle, 28.2%; buffalo, 14.7%; sheep, 18.8%; goat 12.3%	(40)
Peshawar	2003–2004	71.5249° E	34.0151° N	Cattle, buffalo, sheep, donkey, and goat	1,279	Cattle, 20.4%; buffalo, 11.3%; sheep, 12.8%; donkey, 6.4%; goat, 12.1%	(21)
Quetta	2008	66.9750° E	30.1798° N	Cattle and buffalo	790	Cattle, 10.14%; buffalo, 6.99%	(29)
Punjab	2008	72.7097° E	31.1704° N	Cattle	300	66.7%	(30)
Punjab	2008	72.7097° E	31.1704° N	Cattle, buffalo, camel, sheep, and goat	3,400	Cattle, 75.1%; goat, 51.6%; buffalo, 40.08%; camel and sheep, 0%	(18)
Dera Ghazi Khan	2008	70.6455° E	30.0489° N	Cattle	300	36%	(32)
Punjab	2009	72.7097° E	31.1704° N	Cattle and buffalo	3,500	Cattle, 72%; buffalo, 47.3%	(41)
Punjab	2006–2007	72.7097° E	31.1704° N	Cattle	1,000	31.5%	(42)
Attock	2009	72.3609° E	33.7660° N	Sheep and goat	662	Sheep, 43.37%; goat, 41.53%	(43)
KPK	2009	72.3311° E	34.9526° N	Buffalo, cattle, camel, donkey, goat, and sheep	992	Buffalo, 24.13%; cattle, 35.87%; goat, 23.13%; camel, 28.9%; donkey, 4.2%; sheep, 27.3%	(44)
Sahiwal	2010	73.1114° E	30.6682° N	Cattle	300	38.33%	(45)
Lahore	2012	74.3587° E	31.5204° N	Cattle	2,160	65.6%	(46)
Toba Tek Singh	2012	72.652359° E	30.894875° N	Buffalo	1,128	31.21%	(47)
Punjab,	2013	72.7097° E	31.1704° N	Horse, donkey, and mule	450	4%	(36)
KPK	2013	72.3311° E	34.9526° N	Cattle and buffalo	2,529	Cattle, 33.36%; buffalo, 22.58%	(48)
Punjab	2007	72.7097° E	31.1704° N	Cattle and buffalo	1,030	Cattle, 70%; buffalo, 34%	(49)
Sindh	2008–2009	68.5247° E	25.8943° N	Buffalo	1,600	23%	(50)
Punjab	2011–2012	72.7097° E	31.1704° N	Cattle	3,864	39.1%	(17)
Toba Tek Singh	2011–2012	72.652359° E	30.894875° N	Goat	4,020	33.58%	(11)
Sargodha	2012–2013	72.6861° E	32.0740° N	Buffalo and goat	2,400	Buffalo, 84.33%; goat, 86.50%	(51)
Hajira, Rawalakot, Azad Kashmir	2011	73.7810° E	33.7670° N	Cattle, buffalo, sheep, and goat	669	Cattle, 55.45%; buffalo, 51.03%; sheep, 54.66%; goat 48.0%	(52)
Peshawar	2011	71.5249 E	34.0151 N	Goat and sheep	170	Sheep, 66.66%; goat, 73.68%	(2)
Central Punjab	2008	74.2682° E	31.4469° N	Sheep	1,200	64.25%	(53)

#### Prevalence of Ticks in Cows

The prevalence of tick infestation in cows ranged between 10.14 and 89.9%. Ten studies in Punjab reported that 28.2% (40), 31.5% (42), 39.1% (17), 38.33% (45), 66.7% (30), 70% (49), 72% (41), 75.1% (18), and 65.6% (46) of the cows were infested with ticks. These prevalence rates were higher than the 20.4% reported in Peshawar (21). A prevalence of 10.14% was reported in the cows of Quetta (29). The details as shown in Table 3.

Tick infestation prevalence of 35.87% (44) were recorded in the cows of Khyber Pakhtunkhwa, 38.33% in Sahiwal (45), and 65.6% in Lahore from Punjab province (46). The cattle in Hajira, Rawalakot, Azad Kashmir were more infested than other animals, with 55.45% being the highest prevalence recorded (52). 11.73% in a study from 2019 in Islamabad (54), 75% in the District of Karak (10), 63.33% in Multan (3), and 24% in Hyderabad (8).

looseness1In 2017, tick prevalence was recorded in various animal species from Mansehra to Gilgit. Of all the examined cattle, 77.91% were found to be infested with ticks (55). In Baluchistan province (Quetta city) prevalence was 65.96% in the cattle (56). Of the total observed farmed cattle in a study from 2017, 89.9% were infested with ticks in the semiarid and arid agro-ecological zones from Punjab (6). A recent study from 2020 (5) studied tick infestation in various livestock species, finding that 65% of the cows were infested with ticks from Baluchistan. These ticks are responsible of causing different diseases like babesiosis, theileriosis, and anaplasmosis in cattle (57).

#### Prevalence of Ticks in Camels

Of the 44 studies published on ticks as livestock ectoparasites, three examined the prevalence of ticks in camels. A study from 2010 (44) reported that 28.9% of the camels examined were infested with ticks in Khyber Pakhtunkhwa, while 55.56% were found infested in Cholistan (39). More recently, a study from 2020 noted that 47.5% of the examined camels in Balochistan were infested (5).

#### Prevalence of Ticks in Equids

The prevalence of tick infestation in equids was in the range of 0–26.9%. No horse was found to be tick-infested in Lahore (27). The overall reported tick infestation prevalence in donkeys and other animals in Peshawar was 13.37% (21). In 2010, researchers recorded a prevalence of 4.2% in donkeys in Khyber Pakhtunkhwa (44), while the overall tick infestation prevalence in equids in Punjab was 4% (36).

#### Prevalence of Ticks in Buffaloes

The prevalence of ticks in buffaloes varied between regions of Pakistan, as shown in Table 3. The highest prevalence (87.55%) was recorded in Multan (3), and the lowest (3.0%) in Islamabad (54).

The following tick infestation prevalence rates were reported in buffaloes: 5.08% in calves in the Dir District (26); 14.7% (40) and 24.13% (44) in Khyber Pakhtunkhwa; 40.08% (18) and 47.3% (41) in Punjab. The overall prevalence of ticks in different animals in Peshawar, including buffaloes, was 11.30% (21). The detailed prevalence of ticks in other areas is shown in Table 3.

#### Prevalence of Ticks in Sheep and Goats

The prevalence of ticks in goats and sheep varied between regions of Pakistan, as shown in Table 3. In Punjab, it was found that about 18.8% (40) and 64.25% (53) of the sheep and 12.3% (40) of the goats were infested with ticks. A study in Punjab from 2008 recorded a prevalence of 51.6% in goats and 0% in sheep (18). The recorded prevalence in Attock was 43.37% in sheep and 41.53% in goats (43). In 2010, a prevalence of 23.13% was recorded in the goats of Khyber Pakhtunkhwa (44), 33.58% of the goats of the district of Toba Tek Singh (11), and 86.50% of the goats in Sargodha (51). In Hajira, Rawalakot, Azad Kashmir, 54.66% of the sheep and 48.0% of the goats were infested (52), while the respective rates in Peshawar were 66.66% and 73.68% (2).

A 2017 assessment of various animals from Mansehra to Gilgit recorded tick infestation in 81.47% of the sheep and 72.05% of the goats (55), while another study found infestation in 60.0% of the goats and 11.1% of the sheep (6). More recently, studies from 2020 reported tick infestation rates of 30% in sheep and 27.5% in goats in Balochistan (5), and 50% in sheep and 40.30% in goats in Multan (3).

Babesiosis infection holds a massive economic influence due to loss of beef and meat production in infested animals also causes mortality and morbidity in cattle's all over the world (58).

There is a paucity of available data on tick control in small ruminants in Pakistan, partially because farmers prefer cattle over sheep and goats due to their higher economic significance (59). Considering the significant health and environmental risks connected with acaricides such as organophosphorus compounds, formamidines, pyrethroids, macrocyclic lactones, and phenylpyrazoles, their routine administration is the primary technique of ectoparasites management utilized in Pakistan's ruminants (60–62). In addition, grooming, which is the manual plucking of ticks by agricultural workers, is a popular practice in Pakistan for tick management (63). However, only two studies have evaluated the efficiency of acaricidal medicines *in vivo*, and the authors reported that cypermethrin was the most efficient tick control drug in livestock (59, 64). Numerous studies have been conducted to determine the efficiency of different medicines (alone or in combination with antibiotics) in treating ectoparasites, including buparvaquone, diminazene aceturate + imidocarb dipropionate, and oxytetracycline (65).

Ectoparasites are found throughout Pakistan's diverse ecological and topographical zones. Pakistan's diverse landscapes include plains, deserts, forests, and plateaus, extending from the Arabian Sea coast in the south to the mountains in the north. Due to Pakistan's geography inside South Asia's subtropical region (30° N, 70° E), the majority of the country provides favorable climate patterns for parasites especially ticks and other ectoparasites, which may invade a myriad of hosts and transfer illness to human, livestocks, and pet animals (60).

Vector-borne microorganisms and many illnesses are present in the ecology of a range of arthropods, and their prevalence may be rising due to climate change and human-induced arthropod promotion (66). These activities include agricultural methods, communal sports and recreation, tourist and commerce globalization, and forestry incursion, all of which enhance communities' exposure to microorganisms created in these changing environments (67–69). As for the limitation, the review protocol is not registered, which is the primary limitation of the current study.

## Conclusion

Ectoparasites have a detrimental impact on the production and performance of livestock. The data presented in this study revealed a high prevalence of ectoparasite infestations in livestock, including goats, sheep, buffaloes, cattle, camels, and horses. The various ectoparasites transmit a broad spectrum of pathogens to all these animals. Lack of awareness about the magnitude of the problem among owners, unavailability of control systems, and pitiable efficacy of chemical control have contributed to the prevalence of a range of ectoparasites in Pakistan, even after enacting movement control. This review could guide veterinary schools, researchers, and organizations in designing future studies and might support the work of veterinarians, administrators, human well-being care providers, and help nearby countries that might want to help eradicate the ectoparasites from the region.

## Recommendations

- Wakefulness of the local farmers is important for successful control of the ectoparasites.
- Alertness and control programs for livestock farmers concerning the serious and detrimental outcomes of ectoparasite infestations must be launched by various associations.
- Intended treatment of livestock with pesticides must be adapted to each region to lessen the influence of the ectoparasites on the animals fitness.
- Newly acquired animals should be checked and treated before they are introduced into the herd or farmhouse.
- Improved control practices should be applied to lessen the transmission of diseases and increase the livestock yield.
- Proper veterinary facilities and training should be offered as part of the control efforts that should include regular spraying against the ectoparasites.

## Author Contributions

AM, HA, and JC designed the study and provided overall supervision of the current work. MM and RB performed the data collection. MK and UA prepared the maps. MA performed the data analysis. HA and RB drafted the manuscript. SS and JC made critical revisions. All authors read and approved the final manuscript.

## Funding

This study was supported by the National Natural Science Foundation of China (Grant Nos. 81772225 and 81971969 to JC), Key Laboratory of Parasite and Vector Biology, National Health Commission of the People's Republic of China (No. WSBKFKT2017-01), and the Fifth Round of Three-Year Public Health Action Plan of Shanghai (No. GWV-10.1-XK13 to JC). The funders had no role in the study design, the data collection and analysis, the decision to publish, or the preparation of the manuscript.

## Conflict of Interest

The authors declare that the research was conducted in the absence of any commercial or financial relationships that could be construed as a potential conflict of interest.

## Publisher's Note

All claims expressed in this article are solely those of the authors and do not necessarily represent those of their affiliated organizations, or those of the publisher, the editors and the reviewers. Any product that may be evaluated in this article, or claim that may be made by its manufacturer, is not guaranteed or endorsed by the publisher.

## Abbreviations

WHO: World Health Organization
TBD: Tick born diseases
NZDs: Neglected Zoonotic Diseases
DALYs: Disability Adjusted Life Years.
